# Rush Medical College Library and Reading Room

**Published:** 1868-11-15

**Authors:** 


***Rush Medical College Library and Reading Room.***

We are requested to return the thanks of the faculty and students to the editors of medical journals and publishers of medical books who have, with great liberality, furnished their productions to the reading room of the college. Each is appropriately acknowledged at the rooms, and a list has been furnished us for publication, but unfortunately, it is crowded out of the present number.

